# Plant Longevity

**Published:** 1882-04

**Authors:** F. Hildebrand


### RELATIONS OF PLANT LONGEVITY TO GEOGRAPHICAL DISTRIBUTION.

Particularly interesting examples of the evolution of different life-terms are exhibited in the geographical distribution of plants. If we consider the whole earth as to its climate, we shall observe that in a few regions near the equator, that have a uniform climate, plants will grow all the year through without manifesting any periodical preferences. This is the case, for example, east of the Andes in Northern Brazil, in Guiana, and in Java, where the vegetation is green and blooms continuously, where most species become woody, and nearly all live long and bear fruit often ; while the short-lived, once-fruiting species retire to the background. In other tropical regions, where a periodical climate is produced by differences in the moisture of the atmosphere, the long-lived plants prevail, and the ground is so occupied with them till the coming on of the dry season that the short-lived kinds cannot find room upon it. The case is different in those regions where spots become barren of vegetation in consequence of the parching heat. Then, when the rainy season sets in, the annuals quickly spring up between the bulbous and tuberous herbs that are able to keep their places through the drought. The short-lived species are of most importance where a warm season alternates with a cold one, and the warm season lasts long enough for them to go within its term through the whole cycle of their life, from their seed-time to the ripening of their fruit. As the warm season becomes shorter the number of annuals is reduced, until finally, when the summer is not long enough for any of them to perfect their seed, they disappear altogether. Thus the persistent, often-fruiting species gain the monopoly on high mountains and in Arctic regions, but with the difference that in some districts they maintain themselves above ground through the whole year without protection against the climate, while in others they exist through a long period of rest, protected against the injurious effects of the cold by means of their perennial parts under the soil or under the cover of an effective shelter.—F. Hildebrand, *in Popular Science Monthly for March.*

